# Adeno-associated Virus Vector-mediated Interleukin-10 Induction Prevents Vascular Inflammation in a Murine Model of Kawasaki Disease

**DOI:** 10.1038/s41598-018-25856-0

**Published:** 2018-05-15

**Authors:** Jun Nakamura, Sachiko Watanabe, Hiroaki Kimura, Motoi Kobayashi, Tadayoshi Karasawa, Ryo Kamata, Fumitake Usui-Kawanishi, Ai Sadatomo, Hiroaki Mizukami, Noriko Nagi-Miura, Naohito Ohno, Tadashi Kasahara, Seiji Minota, Masafumi Takahashi

**Affiliations:** 1 Division of Inflammation Research, Jichi Medical University, Tochigi, Japan; 2 Division of Rheumatology and Clinical Immunology, Jichi Medical University, Tochigi, Japan; 3 Division of Genetic Therapeutics,Center for Molecular Medicine, Jichi Medical University, Tochigi, Japan; 4 Laboratory for Immunopharmacology of Microbial Products, School of Pharmacy, Tokyo University of Pharmacy and Life Sciences, Hachioji, Tokyo Japan

## Abstract

Kawasaki disease (KD), which is the leading cause of pediatric heart disease, is characterized by coronary vasculitis and subsequent aneurysm formation. Although intravenous immunoglobulin therapy is effective for reducing aneurysm formation, a certain number of patients are resistant to this therapy. Because interleukin-10 (IL-10) was identified as a negative regulator of cardiac inflammation in a murine model of KD induced by *Candida albicans* water-soluble fraction (CAWS), we investigated the effect of IL-10 supplementation in CAWS-induced vasculitis. Mice were injected intramuscularly with adeno-associated virus (AAV) vector encoding IL-10, then treated with CAWS. The induction of AAV-mediated IL-10 (AAV-IL-10) significantly attenuated the vascular inflammation and fibrosis in the aortic root and coronary artery, resulting in the improvement of cardiac dysfunction and lethality. The predominant infiltrating inflammatory cells in the vascular walls were Dectin-2^+^CD11b^+^ macrophages. *In vitro* experiments revealed that granulocyte/macrophage colony-stimulating factor (GM-CSF) induced Dectin-2 expression in bone marrow-derived macrophages and enhanced the CAWS-induced production of tumor necrosis factor-α (TNF-α) and IL-6. IL-10 had no effect on the Dectin-2 expression but significantly inhibited the production of cytokines. IL-10 also inhibited CAWS-induced phosphorylation of ERK1/2, but not Syk. Furthermore, the induction of AAV-IL-10 prevented the expression of TNF-α and IL-6, but not GM-CSF and Dectin-2 at the early phase of CAWS-induced vasculitis. These findings demonstrate that AAV-IL-10 may have therapeutic application in the prevention of coronary vasculitis and aneurysm formation, and provide new insights into the mechanism underlying the pathogenesis of KD.

## Introduction

Kawasaki disease (KD) is an acute febrile multisystem vasculitis that is the most common cause of acquired cardiac disease in children in the developed countries^1^. If untreated, coronary artery aneurysms reportedly occur in approximately 25% of the patients and contribute to the development of adult cardiovascular disease^2–4^. Although the etiology of KD still remains unknown, the administration of intravenous immunoglobulin (IVIG) has been shown to be effective in the acute phase of KD and to reduce the incidence of coronary aneurysm from 25% to 1–5%. However, approximately 10–20% of patients fail to respond to IVIG therapy, and the majority of patients who develop the coronary aneurysms are IVIG-resistants^2–4^. Thus, it is necessary to develop additional therapeutic approaches for treating IVIG-resistant patients and understanding of the underlying mechanisms in the pathogenesis of KD.

Interleukin-10 (IL-10) is a potent immunosuppressive cytokine that inhibits immune responses at different levels by acting on both the innate and acquired immune system^5,6^. In particular, IL-10 inhibits the production of inflammatory cytokines and is implicated in the pathogenesis of inflammatory and autoimmune diseases. The potential therapeutic application of IL-10 in KD treatment has been suggested based on the following observations: first, although the mechanism underlying the effects of IVIG in the pathogenesis of KD is not yet fully understood, recent studies suggest the involvement of the Fc-mediated expansion of regulatory T cells and increased IL-10 secretion^4^. Second, Nagi-Miura *et al*.^7^ examined the sensitivity to *Candida albicans* water-soluble fraction (CAWS)-induced vasculitis, which is a frequently used murine model of KD, among various mouse strains and found that the serum IL-10 levels after the administration of CAWS were elevated in CAWS-resistant CBA/J mice, but not in the CAWS-sensitive strains, suggesting that IL-10 negatively regulates the development of CAWS-induced vasculitis. However, there is currently no information on the efficacy of IL-10 with regard to the development of KD.

In the present study, to explore the effect of IL-10 supplementation in the KD development, we used adeno-associated virus (AAV) vectors because they are efficient vehicles for the systemic and long-term expression of therapeutic proteins^8,9^, and demonstrated that the induction of AAV-mediated IL-10 prevents vascular inflammation and cardiac dysfunction in CAWS-induced vasculitis. We further showed that granulocyte/macrophage colony-stimulating factor (GM-CSF) is essential for the CAWS-induced inflammatory responses in macrophages. These findings indicate that IL-10 has potential applications in the prevention of KD, and provide new insights into the mechanisms underlying the pathogenesis of KD.

## Results

### The AAV-mediated induction of IL-10 improves CAWS-induced cardiac dysfunction and lethality

**Figure 1:**
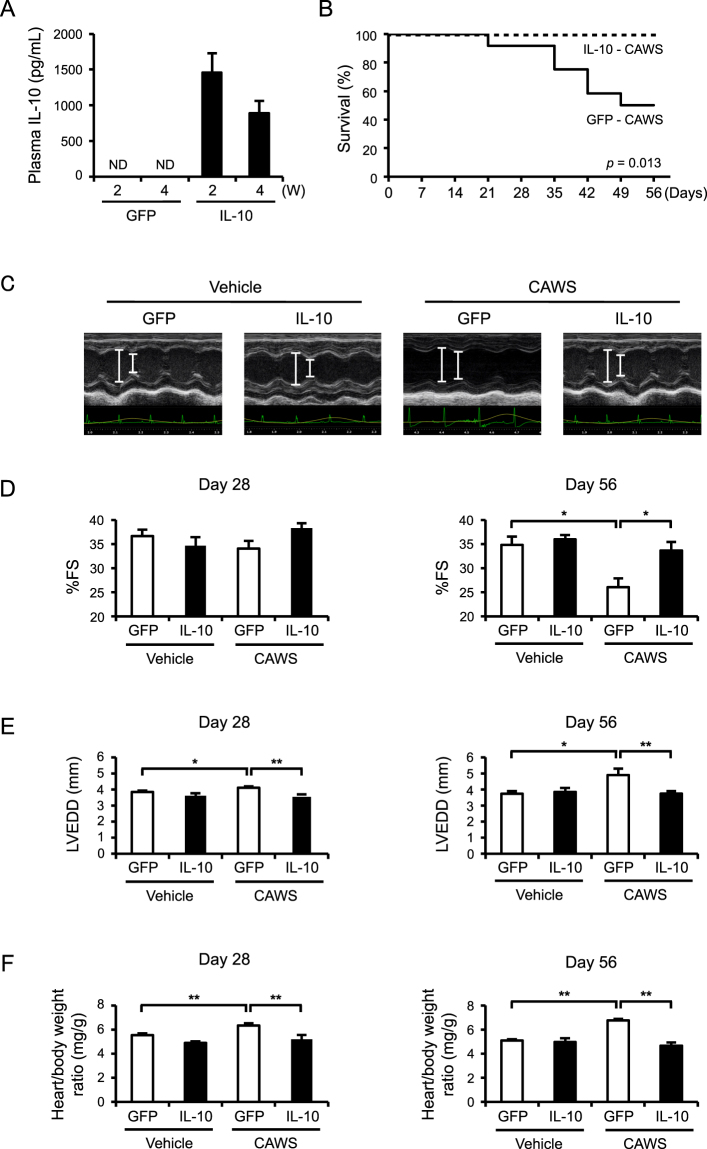
The AAV-mediated induction of IL-10 improves CAWS-induced cardiac dysfunction (**A**) Mice were injected intramuscularly with AAV-GFP (control) or AAV-IL-10. The plasma IL-10 levels were assessed at 2 and 4 weeks after the injection of AAV (n = 11–13). ND indicates not detected. (**B**–**F**) Mice were treated intraperitoneally with CAWS or vehicle at 2 weeks after the AAV injection. The survival of CAWS-treated AAV-GFP and AAV-IL-10 mice was analyzed using Kaplan-Meier method (n = 10–12) (**B**). Echocardiography was performed at 28 and 56 days after CAWS or vehicle treatment. Two-dimensional M-mode echocardiograms at 56 days are shown (**C**). The cardiac function %FS, (**D**) and dimension LVEDD, (**E**), and the heart weight/body weight ratio (**F**) were assessed at 28 and 56 days after CAWS or vehicle treatment (%FS, n = 7–17 [day 28]; n = 4–7 [day 56], LVEDD, n = 7–17 [day 28]; n = 3–6 [day 56], the heart weight/body weight ratio (n = 10–14 [day 28]; n = 5–9 [day 56]). Data are expressed as the mean ± SEM. *p < 0.05, **p < 0.01.

To investigate the effect of IL-10 supplementation *in vivo*, we used AAV vectors and found that the plasma IL-10 levels were markedly elevated in mice treated with AAV-IL-10, whereas no IL-10 was detected in the mice treated with control vectors (AAV-green fluorescent protein [GFP]) (Fig. 1A). Next, to examine the effect of the AAV-mediated induction of IL-10 on KD-related vasculitis, we treated mice with CAWS, 2 weeks after the induction of AAV-IL-10. Plasma IL-10 was not detected in vehicle- and CAWS-treated mice without AAV-mediated IL-10 induction (data not shown). Consistent with a previous report^10^, approximately half of the AAV-GFP mice died at 56 days after CAWS treatment, whereas no AAV-IL-10 mice died during the observation period (Fig. 1B). Echocardiography showed that CAWS treatment induced a marked decrease in the cardiac function (%fraction shortening [FS]) and an increase in cardiac dimension (left ventricle end-diastolic diameter [LVEDD]) in the AAV-GFP mice (Fig. 1C–E). However, the cardiac function and dimension were significantly maintained in the AAV-IL-10 mice. Color Doppler echocardiography showed severe aortic regurgitation at 56 days in all CAWS-treated AAV-GFP mice; however, no aortic regurgitation was detected in the CAWS-treated AAV-IL-10 mice (data not shown). Furthermore, CAWS treatment increased the heart weight/body weight ratio in comparison to vehicle-treated controls, and the ratio was also almost completely restored in the AAV-IL-10 mice (Fig. 1F). There were no adverse events including infection in the AAV-IL-10 mice during the observation period. These results indicate that the AAV-mediated induction of IL-10 improves cardiac dysfunction and lethality in CAWS-induced vasculitis. Because the survival rate of CAWS-treated mice was very low, the following experiments were conducted at 28 days after CAWS treatment.

### The AAV-mediated induction of IL-10 reduces vascular inflammation and fibrosis

**Figure 2:**
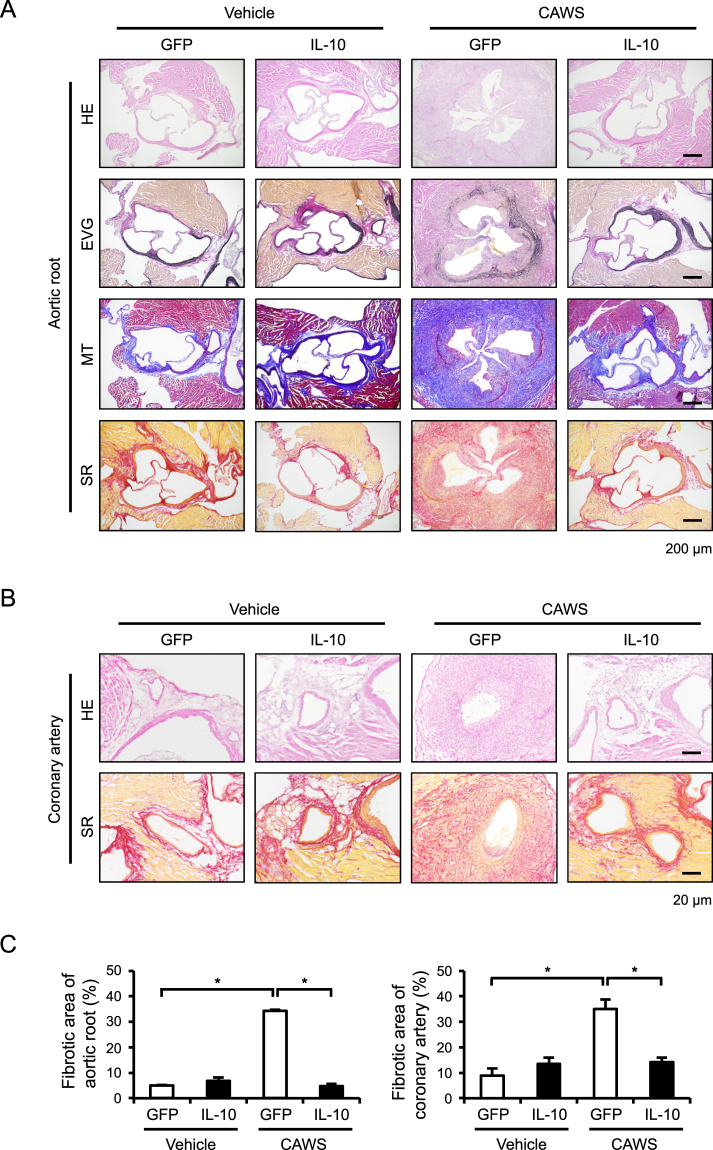
The AAV-mediated induction of IL-10 reduces CAWS-induced vascular inflammation and fibrosis Mice were treated intraperitoneally with CAWS or vehicle, 2 weeks after the injection of AAV-GFP (control) or AAV-IL-10. Heart sections were obtained at day 28 after CAWS or vehicle treatment and stained with HE, EVG, MT, and SR. (**A**,**B**) Representative images of the aortic root and coronary artery are shown. (**C**) The fibrotic area of the aortic root and coronary artery was quantified (n = 3–4). Data are expressed as the mean ± SEM. *p < 0.05.

**Figure 3:**
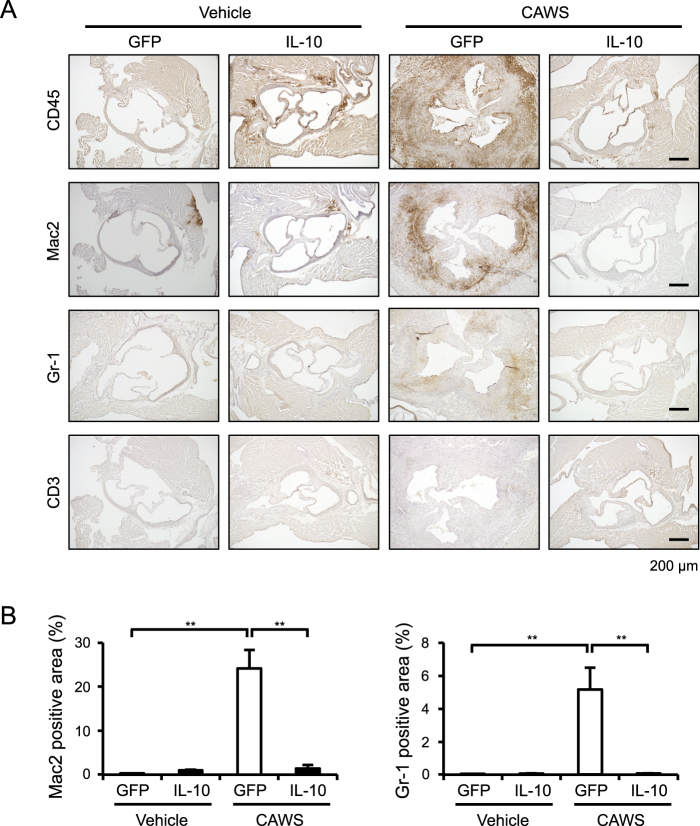
The AAV-mediated induction of IL-10 reduces CAWS-induced inflammatory cell infiltration Mice were treated intraperitoneally with CAWS or vehicle, 2 weeks after the injection of AAV-GFP (control) or AAV-IL-10. (**A**) Heart sections were obtained at day 28 after CAWS or vehicle treatment and subjected to immunohistochemical staining for CD45, Mac2, Gr-1, and CD3. (**B**) The Mac2^+^ and Gr-1^+^ areas were quantified (n = 4–6). Data are expressed as the mean ± SEM. **p < 0.01.

We next performed histological analyses to assess vascular inflammatory and fibrotic responses. Hematoxylin and eosin (HE) and Elastic Van Gieson (EVG) staining revealed prominent inflammatory cell infiltration and the disarrangement of the medial elastic lamina, respectively, at the aortic root and coronary artery of CAWS-treated mice (Fig. 2A,B and Supplementary Figure S1). In addition, Masson’s trichrome (MT) and Sirius Red (SR) staining showed marked fibrotic changes at these sites (Fig. 2A–C). These vascular manifestations were all markedly improved by the AAV-mediated induction of IL-10. To identify the cells that had infiltrated into the vascular walls of the hearts, we performed immunohistochemical staining to detect CD45 (a pan-leukocyte marker), Mac2 (a macrophage marker), Gr-1 (a neutrophils marker), and CD3 (a T cell marker), and found that the infiltration of Mac2^+^ and Gr-1^+^ cells, but not CD3^+^ cells, was significantly increased in the vascular walls of CAWS-treated mice, and that this infiltration was markedly inhibited by the AAV-mediated induction of IL-10 (Fig. 3A and Supplementary Figure S1). A quantitative analysis indicated that macrophages were the predominant inflammatory cells infiltrating in the vascular walls and that they responded to the AAV-mediated induction of IL-10 (Fig. 3B).

### The AAV-mediated induction of IL-10 reduces the expression of inflammatory cytokines and fibrosis-related factors

**Figure 4:**
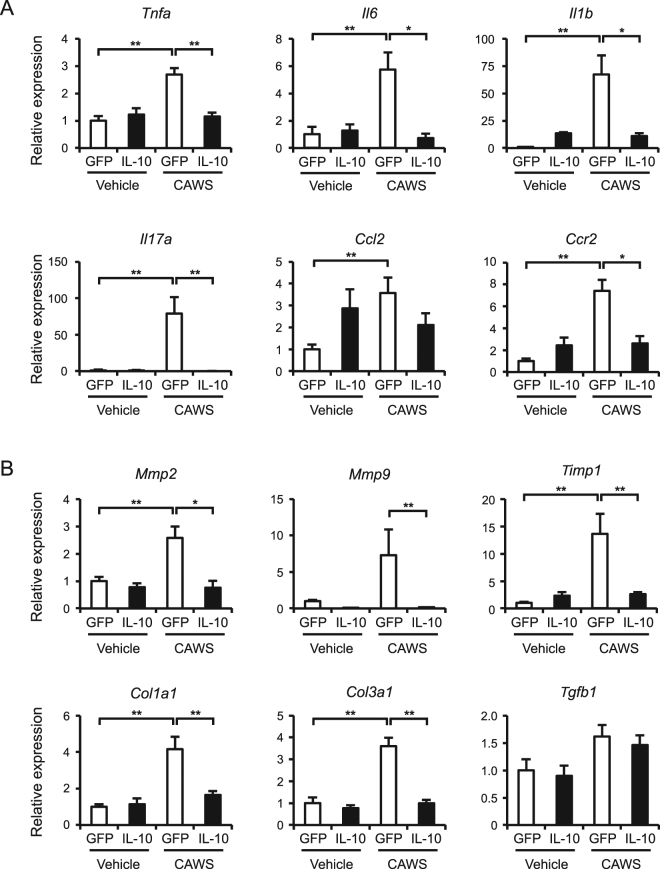
The AAV-mediated induction of IL-10 reduces CAWS-induced inflammatory and fibrotic responses Mice were treated intraperitoneally with CAWS or vehicle, 2 weeks after the injection of AAV-GFP (control) or AAV-IL-10. The heart samples were obtained at day 28 after CAWS or vehicle treatment. The mRNA levels of *Tnfa*, *Il6*, *Il1b*, *Il17a*, *Ccl2*, *Ccr2* (**A**), *Mmp2*, *Mmp9*, *Timp1*, *Col1a1*, *Col3a1*, and *Tgfb1* (**B**) were assessed by a real-time RT-PCR analysis (n = 4–6). Data are expressed as the mean ± SEM. *p < 0.05, **p < 0.01.

To explore the factors involved in the inhibitory effects of IL-10 in CAWS-induced vasculitis, we assessed the expression of inflammatory cytokines and fibrosis-related factors in the heart. A real-time RT-PCR analysis showed that the mRNA levels of TNF-α (*Tnfa*), IL-6 (*Il6*), IL-1β (*Il1b*), IL-17A (*Il17a*), and the inflammatory macrophage marker CCR2 (*Ccr2*) were significantly elevated in the CAWS-treated mice (Fig. 4A). The AAV-mediated induction of IL-10 significantly reduced the expression of these inflammatory cytokines. As expected from the histological findings, the expression levels of fibrosis-related factors, such as MMP-2 (*Mmp2*), TIMP-1 (*Timp1*), and collagen type I and III (*Col1a1* and *Col3a1*), were increased in the CAWS-treated mice and were significantly reduced by the AAV-mediated induction of IL-10 (Fig. 4B). A similar expression pattern was also detected in CCL2 (*Ccl2*) and MMP-9 (*Mmp9*), but it did not reach a statistical significance. These findings suggest that the AAV-mediated induction of IL-10 attenuates inflammatory and fibrotic responses in CAWS-induced vasculitis.

### The AAV-mediated induction of IL-10 reduces infiltration of Dectin-2^+^CD11b^+^ cells

**Figure 5:**
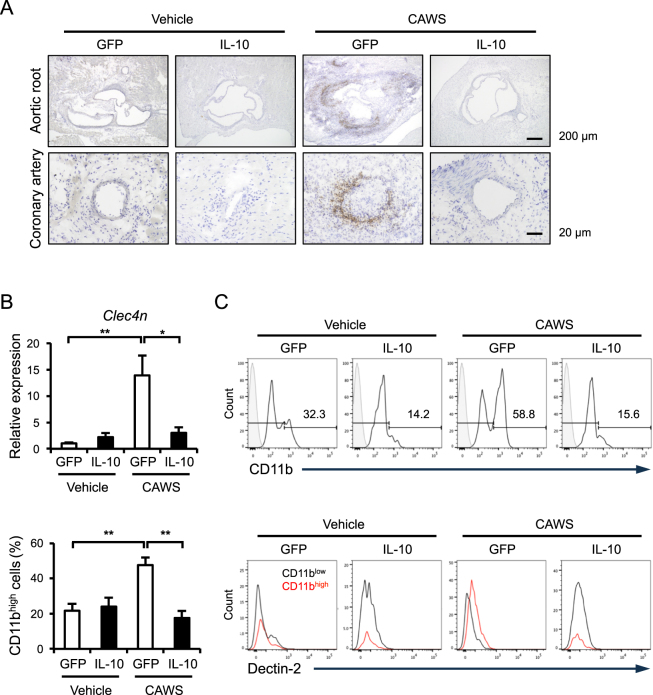
CD11b^+^Dectin-2^+^ cells are involved in the inhibitory effect of AAV-IL-10 Mice were treated intraperitoneally with CAWS or vehicle, 2 weeks after the injection of AAV-GFP (control) or AAV-IL-10. Heart samples were obtained at day 28 after CAWS or vehicle treatment. (**A**) The sections were subjected to immunohistochemical staining for Dectin-2. (**B**) The mRNA levels of *Clec4n* were assessed by a real-time RT-PCR analysis (n = 4–5). (**C**) The population of CD11b^+^Dectin-2^+^CD45^+^ cells was assessed by flow cytometry (n = 7–8). Data are expressed as the mean ± SEM. *p < 0.05, **p < 0.01.

Because CAWS is a mannoprotein-β-glucan complex and has been recently shown to be recognized by C-type lectin receptor Dectin-2^11^, we examined whether Dectin-2 is involved in the inhibitory effects of IL-10 in CAWS-induced vasculitis. An immunohistochemical analysis revealed that Dectin-2 was clearly expressed in the infiltrates at the aortic root and coronary artery of CAWS-treated mice, whereas it was not detected in the CAWS-treated AAV-IL-10 mice (Fig. 5A). Consistent with this result, a real-time RT-PCR analysis showed that Dectin-2 (*Clec4n*) was upregulated in the CAWS-treated mice, and that it was inhibited by AAV-mediated induction of IL-10 (Fig. 5B). To identify the infiltrating cells expressing Dectin-2, the cells were isolated from the heart and analyzed by flow cytometry. The number of CD11b^+^CD45^+^ cells, which was increased in the CAWS-treated mice, was significantly inhibited by the AAV-mediated induction of IL-10 (Fig. 5C). Furthermore, we found that Dectin-2 was more highly expressed in the infiltrating CD11b^high^ cells than it was in the infiltrating CD11b^low^ cells. The population of CD11b^high^Dectin-2^+^ cells was completely reduced by AAV-mediated induction of IL-10.

### IL-10 inhibits the CAWS-induced production of inflammatory cytokines in BMDCs

**Figure 6:**
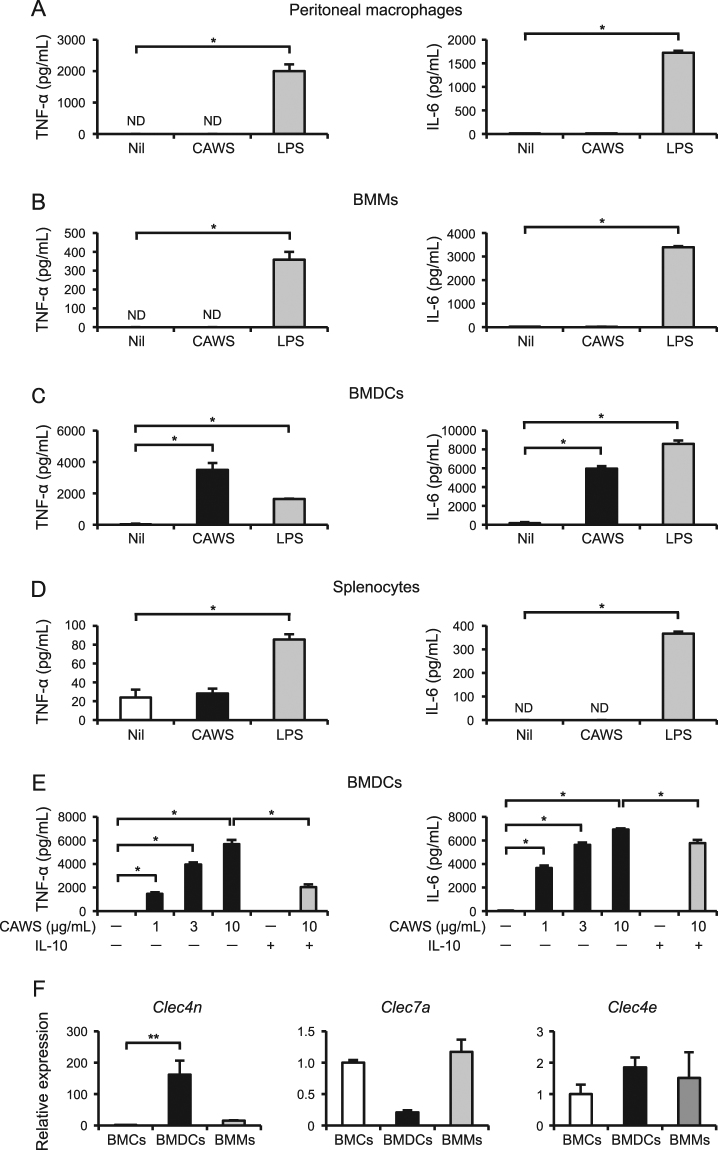
IL-10 inhibits the CAWS-induced production of inflammatory cytokines in BMDCs (**A**–**D**) Primary murine peritoneal macrophages (**A**), BMMs (**B**), BMDCs (**C**), and splenocytes (**D**) were treated with CAWS (10 μg/mL) and LPS (100 ng/mL) for 24 h (peritoneal macrophages) or 48 h (BMMs, BMDCs, and splenocytes). The TNF-α and IL-6 protein levels in the supernatants were assessed (n = 3). (**E**) BMDCs were pretreated with or without IL-10 (40 ng/mL) for 1 h, and then treated with CAWS (1–10 μg/mL) for 48 h. The TNF-α and IL-6 protein levels in the supernatants were assessed (n = 3). (**F)** The mRNA levels of *Clec4n*, *Clec7a*, and *Clec4e* in BMCs, BMDCs, and BMMs were assessed by a real-time RT-PCR analysis (n = 4–6). Data are expressed as the mean ± SEM. *p < 0.05, **p < 0.01.

To determine which cell types could respond to CAWS, we prepared primary murine peritoneal macrophages, bone marrow-derived macrophages (BMMs), bone marrow-derived dendritic cells (BMDCs), and splenocytes *in vitro*. LPS (100 ng/mL), which was used as a positive control, significantly stimulated the production of TNF-α and IL-6 in all cells tested. When stimulated with CAWS (10 μg/mL), the production of TNF-α and IL-6 was detected only in BMDCs, but not in peritoneal macrophages, BMMs, and splenocytes (Fig. 6A–D). The inflammatory cytokine production in the BMDCs was dose-dependent (1–10 μg/mL), and was partially but significantly inhibited by IL-10 (40 ng/mL) (Fig. 6E). Furthermore, the Dectin-2 specific-agonist furfurman induced the production of TNF-α and IL-6 in BMDCs, and IL-10 completely and partially inhibited the production of TNF-α and IL-6, respectively (Supplementary Figure S2). Consistent with these findings, the expression levels of *Clec4n*, but not other C-type lectin receptors (*Clec7a* and *Clec4e* encoding Dectin-1 and Mincle, respectively), in BMDCs were much higher than those in freshly isolated bone marrow cells (BMCs) or BMMs (Fig. 6F).

### GM-CSF enhances the CAWS-induced production of inflammatory cytokines

**Figure 7:**
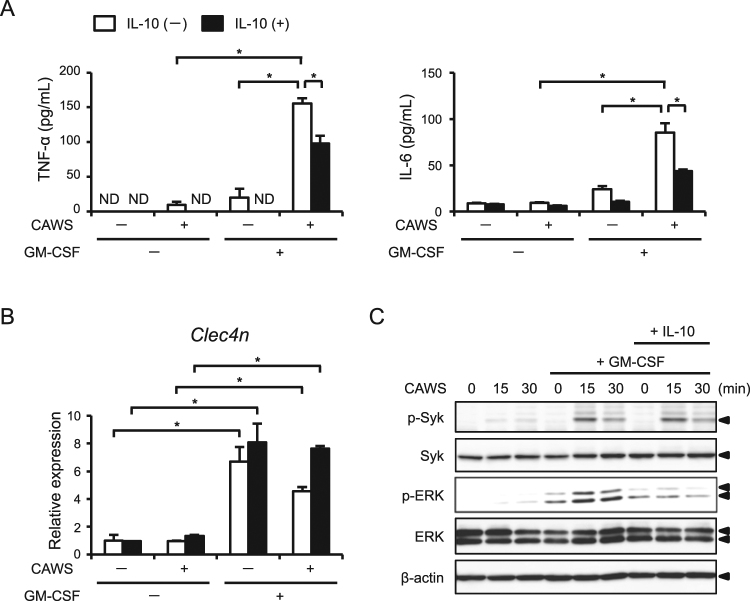
GM-CSF enhances the CAWS-induced inflammatory cytokine production in BMMs BMMs were pretreated with or without IL-10 (40 ng/mL) for 1 h, and then treated with CAWS (10 μg/mL) and/or GM-CSF (1 ng/mL) for 48 h. (**A**) The TNF-α and IL-6 protein levels in the supernatants were assessed (n = 3). (**B**) The mRNA levels of *Clec4n* were assessed by a real-time RT-PCR analysis (n = 3). Data are expressed as the mean ± SEM. *p < 0.05. (**C**) BMMs were treated with or without GM-CSF (1 ng/mL) for 48 h. The cells were pretreated with or without IL-10 (40 ng/mL) for 1 h, and then treated CAWS (10 μg/mL) for the indicated periods. Cell lysates were prepared and analyzed by Western blotting with antibodies against Syk, p-Syk, ERK1/2, p-ERK1/2, and β-actin.

BMDCs and BMMs were generated from murine BMCs in the presence of GM-CSF and macrophage colony-stimulating factor (M-CSF), respectively. In addition, Stock *et al*.^12^ recently identified GM-CSF as one of the cytokines involved in the initiation of cardiac inflammation in a murine model of vasculitis, similar to our CAWS-induced model. Thus, we assumed that GM-CSF is a determining factor of the sensitivity of macrophages to CAWS stimulation. To test this assumption, we stimulated BMMs with CAWS in the presence or absence of GM-CSF (1 ng/mL), and then assessed the cytokine production. GM-CSF alone modestly induced the production of TNF-α and IL-6, whereas it markedly enhanced the production of these cytokines in response to CAWS (Fig. 7A). The enhanced production of these cytokines was significantly prevented by IL-10 treatment. GM-CSF also significantly upregulated the expression of *Clec4n*, but CAWS or IL-10 did not influence its expression (Fig. 7B). These results suggest that GM-CSF enhances the sensitivity of macrophages to CAWS.

Because IL-10 had no effect on the expression of *Clec4n*, we explored the downstream signaling affected by IL-10. Previous studies demonstrated that Syk and the subsequent extracellular-regulated kinase1/2 (ERK1/2) signaling pathway are involved in the Dectin-2-mediated production of inflammatory cytokines^13,14^. Therefore, we next performed Western blotting to assess the activation of Syk and ERK1/2. CAWS treatment induced the phosphorylation of Syk, ERK1/2, JNK, and p38 in GM-CSF-treated BMMs (Fig. 7C and Supplementary Figure S3). Of note, IL-10 inhibited the CAWS-induced phosphorylation of ERK1/2, but not Syk, JNK, and p38. These results suggest that IL-10 inhibits the production of inflammatory cytokines through—at least in part—the inhibition of ERK1/2 activation.

### IL-10 inhibits the expression of TNF-α and IL-6, but not Dectin-2 and GM-CSF, at the early phase of CAWS-induced vasculitis

**Figure 8:**
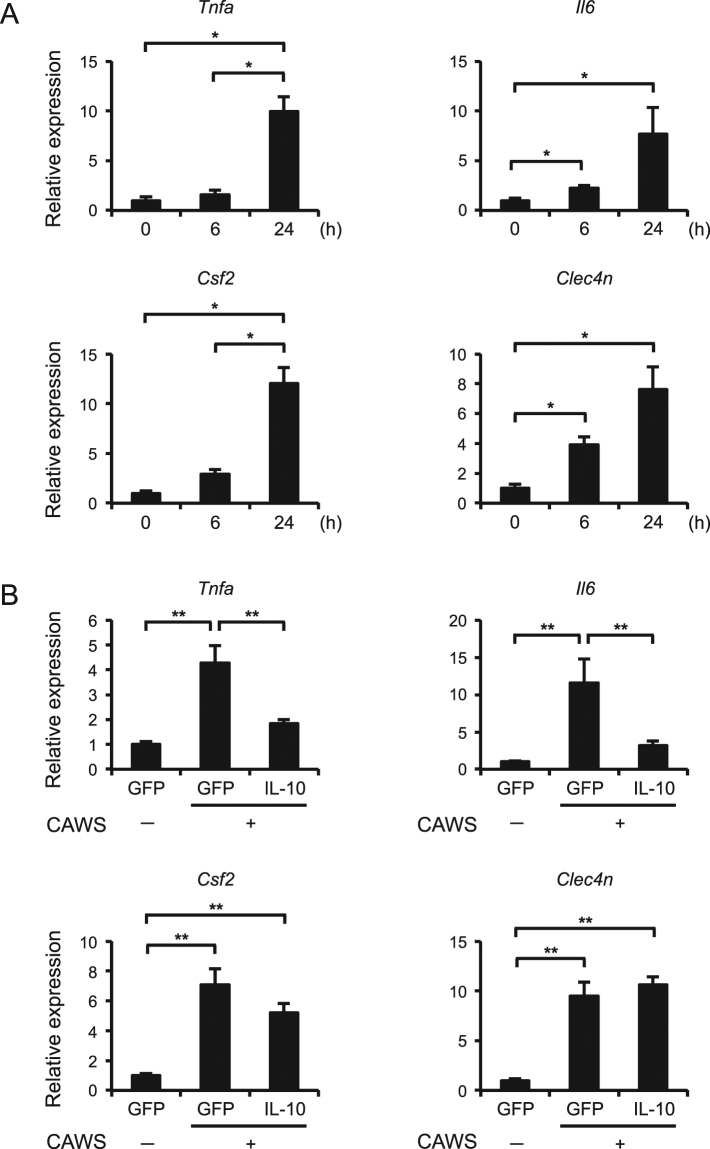
IL-10 inhibits the expression of TNF-α and IL-6, but not GM-CSF and Dectin-2, in the early phase of CAWS-induced vasculitis (**A**) Mice were treated intraperitoneally with a single administration of CAWS, and heart samples were obtained at 6 and 24 h after the administration. The mRNA levels of *Tnfa*, *Il6*, *Csf2*, *Clec4n* were assessed by a real-time RT-PCR analysis (n = 4). (**B)** Mice were treated intraperitoneally with a single administration of CAWS, 2 weeks after the injection of AAV-GFP (control) or AAV-IL-10. Heart samples were obtained at 24 h after the administration. The mRNA levels of *Tnfa*, *Il6*, *Csf2*, *Clec4n* were assessed by a real-time RT-PCR analysis (n = 8–10). Data are expressed as the mean ± SEM. *p < 0.05, **p < 0.01.

To investigate whether Dectin-2 and GM-CSF could be involved in CAWS-induced vasculitis, we analyzed them at the early phase after single CAWS treatment. The expression of *Tnfa*, *Il6*, GM-CSF (*Csf2*), and *Clec4n* was clearly increased at 24 h after CAWS treatment (Fig. 8A). Consistent with the *in vitro* data, the AAV-mediated induction of IL-10 significantly inhibited the expression of *Tnfa* and *Il6*, but not *Csf2* and *Clec4n* (Fig. 8B). These results suggest that IL-10 inhibits the downstream signaling pathway of GM-CSF and Dectin-2.

## Discussion

KD is an acute vasculitis that predominantly targets the medium-sized arteries, particularly the coronary artery, and which leads to the development of coronary artery aneurysm if untreated with IVIG. Although IVIG therapy significantly reduces the occurrence of coronary aneurysm and the morbidity and mortality associated with KD, some patients are resistant to this therapy; thus, additional therapies are still needed^2,15^. In the present study, we demonstrated that IL-10 supplementation by AAV gene delivery almost completely prevented inflammatory responses, such as inflammatory cell infiltration and the expression of cytokines, and the subsequent development of cardiac fibrosis and dysfunction in CAWS-induced vasculitis, a murine model of KD. Furthermore, we found that GM-CSF markedly strengthened the sensitivity of macrophages to CAWS through the upregulation of Dectin-2, which is necessary for CAWS-driven inflammation. IL-10 did not inhibit the GM-CSF-induced Dectin-2 expression but did inhibit the downstream ERK1/2 activation and the production of inflammatory cytokines. These results indicate that IL-10 has potential application in the prevention of KD-related vasculitis, and provide new insights into the underlying mechanisms of this disease.

Epidemiological studies suggest seasonal variation in the occurrence of KD, indicating that environmental factors may contribute to the susceptibility and severity of KD^16^; however, no etiological factors had been identified. Recently, Rodo *et al*.^17^ reported that tropospheric wind patterns from northeastern China are associated with the incidence of KD in Japan, and identified the *Candida* species as the dominant fungus in the tropospheric dust: this suggests that the *Candida* species may be an etiological factor. To date, three murine models are most commonly used to study the pathogenesis of vasculitis in KD: the administration of CAWS, *Lactobacillus casei* cell-wall extract (LCWE), and a synthetic Nod1 ligand^18–20^. Although the vasculitis seen in these murine models is similar to KD, the findings by Rodo *et al*. suggest that CAWS-induced vasculitis is appropriate for studying the pathogenesis of KD. Nagi-Miura *et al*.^7,20^ used this model to assess the sensitivity to CAWS-induced vasculitis among various murine strains and found that the incidence of vasculitis was 100% in C57BL/6, C3H/HeN, and DBA/2 mice, but only 10% in CBA/J mice. Intriguingly, the serum IL-10 levels were elevated after the administration of CAWS in CAWS-resistant CBA/J mice, but not in the CAWS-sensitive strains. These findings prompted us to evaluate the efficacy of IL-10 supplementation in CAWS-mediated vasculitis. Furthermore, clinical studies have previously suggested the association between the severity of KD and the IL-10 levels or polymorphism^21–24^. In the present study, we clearly showed that the AAV-mediated induction of IL-10 almost completely inhibits vascular inflammation and cardiac remodeling in CAWS-induced vasculitis.

Clinical and experimental studies implicated a number of inflammatory cytokines are implicated in the pathogenesis of KD^2,15^. Among these, TNF-α is one of the most important cytokines in the development of KD. Indeed, infliximab, a chimeric murine/human IgG1 monoclonal antibody against TNF-α, has been shown to be effective in treating patients with KD refractory to initial IVIG therapy, with regard to the resolution of fever and inflammatory marker levels; however, this treatment did not change the outcome of coronary artery disease^25^. Another potent inflammatory cytokine, IL-1β, has been shown to be involved in the vascular inflammation and aneurysm formation in a murine model of LCWE-induced vasculitis^19,26^. In addition, several case reports have described the efficacy of IL-1 receptor antagonist anakinra in IVIG-resistant KD patients^27–29^. These findings indicate that multiple inflammatory cytokines participate in the pathogenesis of KD; thus, the inhibition of a single inflammatory pathway may be insufficient for treating KD-related vasculitis. IL-10 is a potent anti-inflammatory cytokine that inhibits the production of multiple inflammatory cytokines, including TNF-α and IL-1β^5,6^. Thus, we assume that IL-10 is a potential candidate for anti-inflammatory therapy in KD-related vasculitis. With respect to clinical application of IL-10, a number of clinical trials regarding IL-10 have been conducted. Supplementation of recombinant human IL-10 in psoriasis patients provided some clinical improvement, whereas it failed to improve Crohn’s disease or rheumatoid arthritis^6,30,31^. The serious side effects that occur in IL-10 therapy are anemia and thrombocytopenia. Although the reason why clinical trials of IL-10 supplementation in autoimmune diseases are largely ineffective remains unclear, it is considered that the effects of IL-10 seem to be quite complex and further clinical and experimental studies are needed for clinical application of IL-10.

Previous investigations suggest that CD11c^+^ macrophages contribute to the pathogenesis of KD in LCWE- and Nod1 ligand-induced KD models^18,19,26^. In the present study, although we did not observe an increase in the CD11c^+^ cells in the hearts of CAWS-induced vasculitis (data not shown), we showed that CD11b^high^Dectin-2^+^ macrophages may be responsible for the vasculitis of KD because Dectin-2 is considered to be the receptor for CAWS^11^. Furthermore, GM-CSF, which induces Dectin-2 in macrophages, is upregulated at the early phase of CAWS-induced vasculitis. In this regard, Stock *et al*.^12^ recently demonstrated that the initial production of GM-CSF by cardiac fibroblasts primes cardiac inflammation in CAWS-induced vasculitis, and GM-CSF blockade markedly improves inflammatory status. Because we also previously showed the essential role of cardiac fibroblasts in NLRP3 inflammasome activation after cardiac ischemia-reperfusion^32^, the role of cardiac fibroblasts in KD-related vasculitis remains to be examined in the future. Based on these findings, we propose the following mechanism: CAWS induces GM-CSF in cardiac fibroblasts, which in turn upregulates Dectin-2 and activates the subsequent signaling pathways, such as Syk and ERK1/2, leading to the production of inflammatory cytokines by macrophages. IL-10 exerts anti-inflammatory effects through—at least in part—inhibiting the activation of EKR1/2 signaling and its downstream pathway.

Several limitations of this study should be noted. First, we clearly showed the preventive effect of IL-10 supplementation on vascular inflammation and lethality in CAWS-induced vasculitis; however, the therapeutic efficacy of IL-10 in established vasculitis remains to be examined. Second, although we showed that the anti-inflammatory effects of IL-10 is partially mediated through EKR1/2 signaling, its precise mechanism still need to be determined. Thus, further investigations are necessary to elucidate the mechanism underlying KD-related vasculitis and the efficacy of IL-10 in this disease.

In conclusion, this study provides two important findings. First, the AAV-mediated induction of IL-10 prevents vascular inflammation and fibrosis, and lethality in a murine model of KD. Second, GM-CSF-upregulated Dectin-2 is essential for the CAWS-induced inflammatory responses in macrophages. Our results suggest the potential application of IL-10 supplementation in the patients with IVIG-resistant KD and provide new insights into the mechanisms underlying the pathogenesis of KD.

## Materials and Methods

### Animal protocols

All experiments in this study were approved by the Use and Care of Experimental Animals Committee of the Jichi Medical University Guide for Laboratory Animals (permit number 17152) and were conducted in accordance with the guidelines of Jichi Medical University. DBA/2 mice (male, 8–weeks–old) were purchased from Japan SLC, Inc. (Tokyo, Japan). Mice were housed (4/cage, RAIR HD ventilated Micro-Isolator Animal Housing Systems, Lab Products, Seaford, DE) in an environment maintained at 23 ± 2 °C with *ad libitum* access to food and water under a 12-h light and dark cycle with lights on from 7:00 to 19:00.

CAWS-induced vasculitis was produced as described previously^7,20^. Briefly, CAWS was prepared from *C. albicans* strain NBRC1385. DBA/2 mice were treated intraperitoneally with CAWS (1 mg/mouse/day) for 5 consecutive days and analyzed at 28 and 56 days after CAWS treatment. For the early-phase analysis, DBA/2 mice were treated with a single administration of CAWS (4 mg/mouse) and analyzed after 6 and 24 h. To induce the overexpression of IL-10 *in vivo*, we prepared AAV serotype 1 encoding murine IL-10 (AAV–IL-10) and control vectors (AAV-GFP)^9,33^. Two weeks prior to CAWS treatment, the AAV vectors (a vector dose of 2 × 10^11^ genome copies/mouse, 100 μL/mouse) were injected into the gastrocnemius muscle of mice.

### Real-time RT-PCR analysis

Total RNA was prepared from the heart using ISOGEN (Nippon Gene Co., Ltd., Toyama, Japan) according to the manufacturer’s instructions. A real-time RT-PCR analysis was performed using a Takara TP960 PCR Thermal Cycler Dice Detection System (Takara Bio Inc, Shiga, Japan) to detect mRNA expression. The primers used in this study were listed (Supplementary table). The expression levels of each target gene were normalized by subtracting the corresponding glyceraldehyde-3-phosphate dehydrogenase (*Gapdh*) threshold cycle (C_T_) values; normalization was carried out using the ΔΔC_T_ comparative method.

### Histology and immunohistochemistry

Hearts were embedded and frozen in Tissue-Tek O.C.T. compound (Sakura Finetechnical Co. Ltd., Tokyo, Japan) after being dehydrated with 20% sucrose, and were cut into 6-μm-thick sections (Leica CM1850; Leica Microsystems, Germany). Cross-sections of the aortic root and coronary artery specimens were stained with HE, EVG, MT, and SR. An immunohistochemical analysis was performed to detect the pan-leukocyte marker CD45 (BD Bioscience), macrophage marker Mac2 (CEDARLANE, Canada), neutrophil marker Gr-1 (Thermo Fisher Scientific), T cell marker CD3 (Thermo Fisher Scientific), and Dectin-2 (Bio-Rad). Isotype-matched IgG (Vector Laboratories) was used as a negative control. The stained sections were photographed using a fluorescence microscope (FSX-100; Olympus, Tokyo, Japan), and quantitatively analyzed using the Adobe Photoshop CS4 software (Adobe System Inc., CA).

### Echocardiography

Transthoracic echocardiography was performed using a digital ultrasound system (Vevo2100 imaging System, Visual Sonics, Toronto, Canada), as previously described^9,34^. Briefly, after anesthetization by inhalation of 1.5% isoflurane, two-dimensional targeted M-mode echocardiograms were obtained along the short axis of the LV at the level of the papillary muscles, and at least three consecutive beats were evaluated. The LVEDD and LV end-systolic diameter (LVESD), defined as the phases in which the area of the LV was smallest and largest, respectively, were obtained. %FS was calculated using the standard formula.

### Cytokine measurement

The levels of IL-10, IL-6, and TNF-α were assessed using a mouse enzyme-linked immunosorbent assay kit (R&D Systems, Minneapolis, MN), according to the manufacturer’s instructions.

### Flow cytometry analysis

Cells were analyzed by flow cytometry, as described previously^9^. The cells were incubated with rat anti-Dectin-2 (Bio-Rad) as the primary antibody and allophycocynain (APC)-conjugated goat anti-rat as the secondary antibody. The cells were also double-labeled with the following antibodies: BV421-conjugated anti-CD45 and BV510-conjugated anti-CD11b antibodies (eBiosceince, San Diego, CA). The cells were examined by flow cytometry (FACS Verse; BD Biosciences); the analysis was performed using FlowJo software (version 10; Tree Star, Inc., San Carlos, CA). Isotype control antibodies were used as negative controls to exclude nonspecific background staining and 7-AAD (BD Biosciences) was used for labeling and excluding dead cells.

### Western blotting

The expression of Syk, ERK1/2, JNK, p38, and their phosphorylated forms (p-Syk, p-ERK1/2, p-JNK, and p-p38), and β-actin was analyzed by Western blotting^9^. The antibodies used in this study were purchased from Cell Signaling Technology, Inc. (Danvers, MA), Santa Cruz Biotechnology (Dallas, TX), and Sigma. The expression level of β-actin served as an internal control for protein loading.

### Cell cultures and *in vitro* experiments

Primary BMCs and splenocytes were isolated from C57BL/6 J mice (Japan SLC, Inc.), and cultured in RPMI-1640 medium supplemented with 10% fetal calf serum (FCS). BMMs and BMDCs were generated from BMCs using 15% conditioned medium of L929 cells (ATCC, Rockville, MD) and recombinant murine GM-CSF (PeproTech Inc., NJ), respectively^35,36^. Murine peritoneal macrophages were prepared from mice using the thioglycolate elicitation method. Recombinant murine IL-10 and furfurman were purchased from PeproTech Inc. and InvivoGen (San Diego CA), respectively. All reagents were obtained from Sigma unless otherwise specified.

### Statistical analysis

The data were expressed as the mean ± standard error of the mean (SEM). For comparisons between multiple groups, the significance of differences in between-group median was determined by Kruskal-Wallis test. Comparisons of unpaired outcomes were assessed by the Mann-Whitney test. The survival of mice was analyzed by the Log-rank test. All analyses were performed using the GraphPad Prism software program (version 7, San Diego, CA). *P*-values of <0.05 were considered statistically significant.

## Electronic supplementary material

Supplementary information
