# Supplementary material for: Adeno-associated Virus Vector-mediated Interleukin-10 Induction Prevents Vascular Inflammation in a Murine Model of Kawasaki Disease
Source: Sci Rep. 2018 May 15;8:7601. doi: 10.1038/s41598-018-25856-0 (PMC5953966; doi:10.1038/s41598-018-25856-0)
Supplement: Supplementary file 1 — Supplementary information [file 41598_2018_25856_MOESM1_ESM.pdf]

Supplementary information

Adeno-associated Virus Vector-mediated Interleukin-10 Induction Prevents Vascular Inflammation in a Murine Model of Kawasaki Disease

Jun Nakamura[1,2], Sachiko Watanabe[1], Hiroaki Kimura[1], Motoi Kobayashi[1], Tadayoshi Karasawa[1], Ryo Kamata[1], Fumitake Usui-Kawanishi[1], Ai Sadatomo[1], Hiroaki Mizukami[3], Noriko Nagi-Miura[4], Naohito Ohno[4], Tadashi Kasahara[1], Seiji Minota[2], Masafumi Takahashi[1]*

[1]Division of Inflammation Research, [3]Division of Genetic Therapeutics, Center for Molecular Medicine, Jichi Medical University, Tochigi, Japan;
[2]Division of Rheumatology and Clinical Immunology, Jichi Medical University, Tochigi, Japan;
[4]Laboratory for Immunopharmacology of Microbial Products, School of Pharmacy, Tokyo University of Pharmacy and Life Sciences, Hachioji, Tokyo, Japan

*Correspondence:
Masafumi Takahashi, MD, PhD
Division of Inflammation Research
Center for Molecular Medicine
Jichi Medical University
3311-1 Yakushiji, Shimotsuke, Tochigi 329-0498, Japan
Tel/ Fax: +81-285-58-7446/+81-285-44-5365
E-mail: masafumi2@jichi.ac.jp

Supplementary table. List of the primers used in this study

**Gene**	**Forward**	**Reverse**
Tnfa	5'-CCCCAAAGGGATGAGAAGTTC-3'	5'-GCTTGTCACTCGAATTTTGAGAA-3'
Il6	5'-ACAACCACGGCCTTCCCTACTT-3'	5'-CACGATTTCCCAGAGAACATGTG-3'
Il1b	5'-TGAAGTTGACGGACCCCAAA-3'	5'-TGATGTGCTGCTGTGAGATT-3'
Il17a	5'-ACGCGCAAACATGAGTCCAG-3'	5'-CTCAGCAGCAGCAACAGCATC-3'
Ccl2	5'-GGCTCAGCCAGATGCAGTTAAC-3'	5'-GCCTACTCATTGGGATCATCTTG-3'
Ccr2	5'-CCTGCAAAGACCAGAAGAGGG-3'	5'-GAGATGTTGATAGTATGCCGTGGA-3'
Mmp2	5'-GACATACATCTTTGCAGGAGACAAG-3'	5'-TCTGCGATGAGCTTAGGGAAA-3'
Mmp9	5'-CCTGGAACTCACACGACATCTTC-3'	5'-TGGAAACTCACACGCCAGAA-3'
Timp1	5'-GTAAGGCCTGTAGCTGTGCC-3'	5'-AGGTGGTCTCGTTGATTTCT-3'
Col1a1	5'-GAGCGGAGAGTACTGGATCG-3'	5'-GTTCGGGCTGATGTACCAGT-3'
Col3a1	5'-CCCAACCCAGAGATCCCATT-3'	5'-GAAGCACAGGAGCAGGTGTAGA-3'
Tgfb1	5'-CTCCCGTGGCTTCTAGTGC-3'	5'-GCCTTAGTTTGGACAGGATCTG-3'
Clec4n	5'-AAGCGGAGCAGAATTTCATCA-3'	5'-CCATTTGCCATTACCTTGTGGA-3'
Clec7a	5'-TGCTTTGTGGTAGTAGTGGTTGCT-3'	5'-TGTGGGCTTGTGGTTCTCTTT-3'
Clec4e	5'-CAGTGGCAATGGGTGGATGATTAC-3'	5'-AGTCCCTTATGGTGGCACAGTC-3'
Csf2	5'-TCGTCTCTAACGAGTTCTCCTT-3'	5'-CGTAGACCCTGCTCGAATATCT-3'
Gapdh	5'-TGTGTCCGTCGTGGATCTGA-3'	5'-TTCGTGTTGAAGTCGCAGGAG-3'

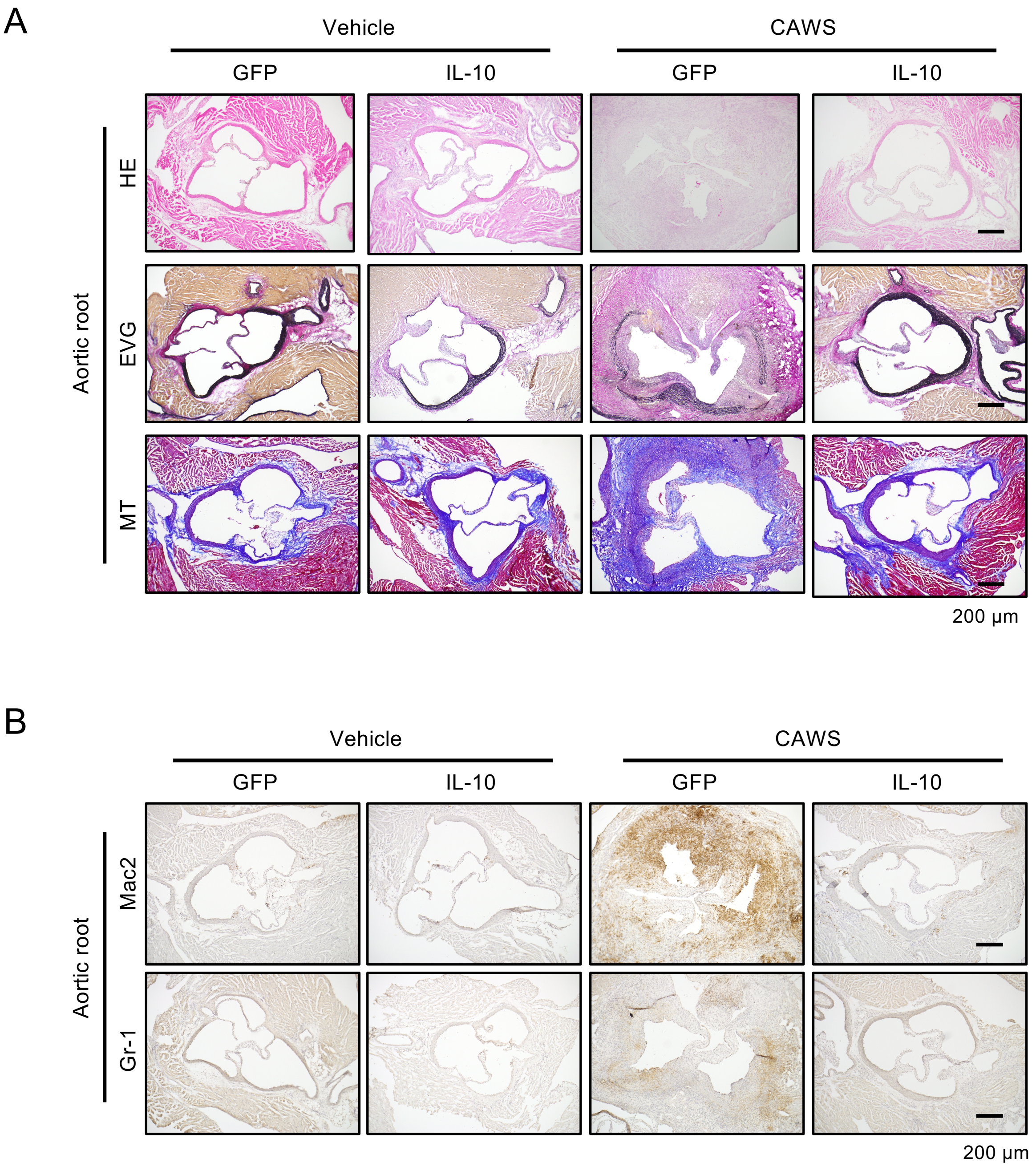

Supplementary figure S1. Histological analysis of the heart sections at 56 days after CAWS treatment

Mice were treated intraperitoneally with CAWS or vehicle, 2 weeks after the injection of AAV-GFP (control) or AAV-IL-10. Heart sections were obtained at day 56 after CAWS or vehicle treatment. (A) HE, EVG, and MT staining. (B) Mac2 and Gr-1 immunohistochemical staining. Representative images of the aortic root and coronary artery are shown.

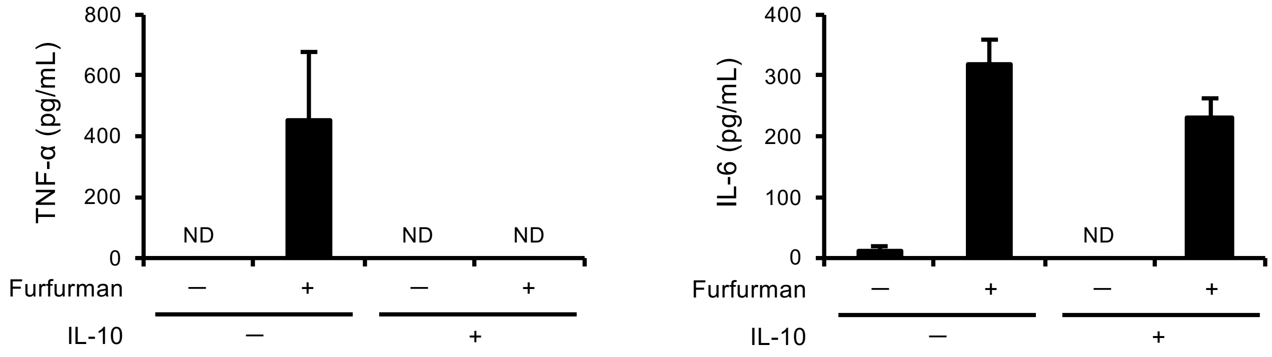

Supplementary figure S2. Effect of furfurman on the production of TNF-α and IL-6 in BMDCs

BMDCs were pretreated with or without IL-10 (40 ng/mL) for 1 h, and then treated with furfurman (1–10 μg/mL) for 48 h. The TNF-α and IL-6 protein levels in the supernatants were assessed (n = 3).

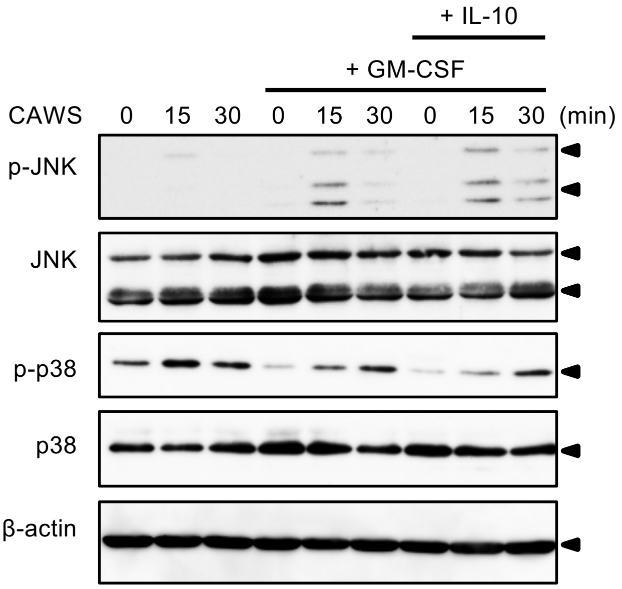

Supplementary figure S3. Effect of CAWS on the activation of JNK and p38
BMMs were treated with or without GM-CSF (1 ng/mL) for 48 h. The cells were pretreated with or without IL-10 (40 ng/mL) for 1 h, and then treated CAWS (10 μg/mL) for the indicated periods. Cell lysates were prepared and analyzed by Western blotting with antibodies against JNK, p-JNK, p38, and p-p38.

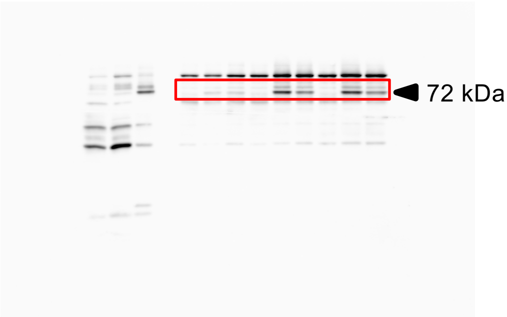

IB: anti-p-Syk

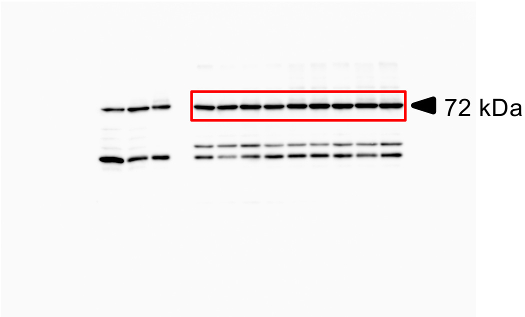

IB: anti-Syk

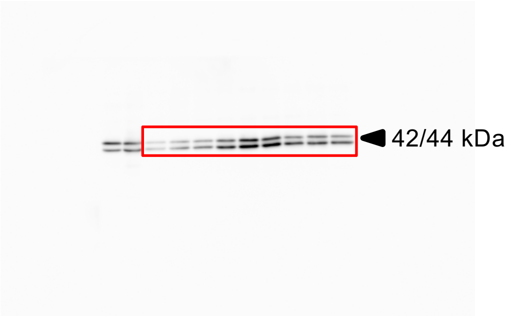

IB: anti-p-ERK1/2

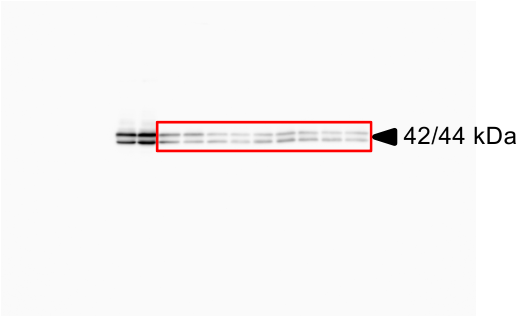

IB: anti-ERK1/2

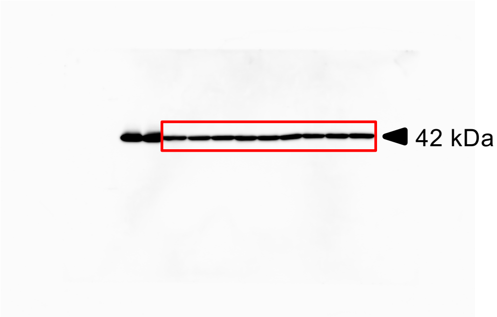

IB: anti-β-actin

Supplemental figure S4. Uncropped images of Western blotting in Figure 7C
Cell lysates were prepared and separated by SDS-PAGE and transferred to PVDH membrane. The membrane was immunoblotted with anti-p-Syk (72 kDa), Syk (72 kDa), p-ERK1/2 (42/44 kDa), ERK1/2 (42/44 kDa), or β-actin (42 kDa) antibodies. The cropped parts indicated by the boxes are shown in Figure 7C.

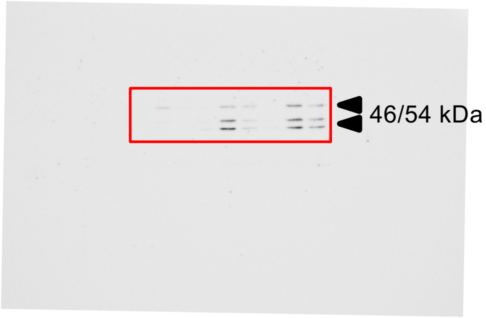

IB: anti-p-JNK

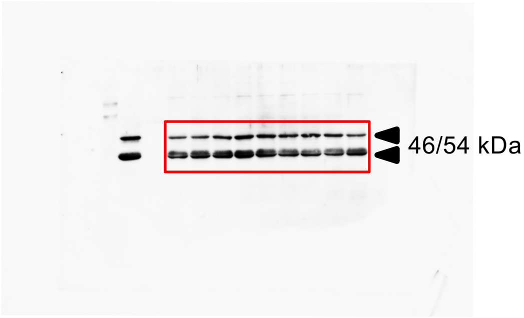

IB: anti-JNK

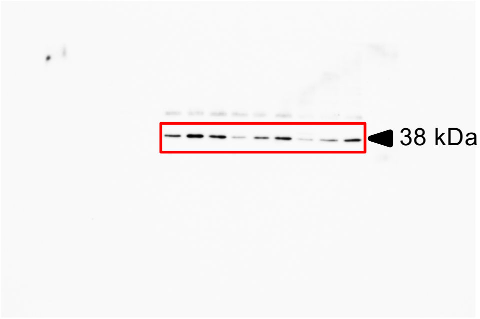

IB: anti-p-p38

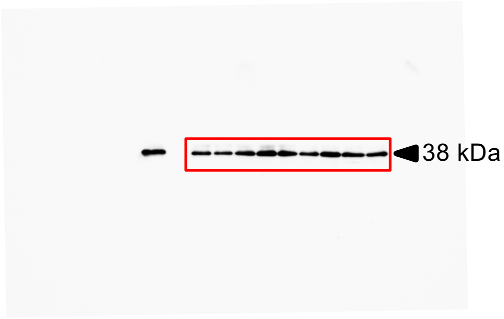

IB: anti-p38

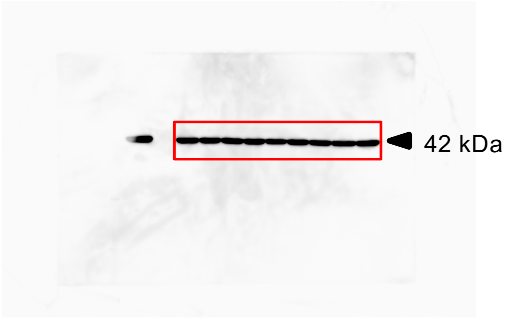

IB: anti-β-actin

Supplemental figure S5. Uncropped images of Western blotting in Supplemental figure S3
Cell lysates were prepared and separated by SDS-PAGE and transferred to PVDH membrane. The membrane was immunoblotted with anti-p-JNK (46/54 kDa), JNK (46/54 kDa), p-p38 (38kDa), p38 (38 kDa), or β-actin (42 kDa) antibodies. The cropped parts indicated by the boxes are shown in Supplemental figure S3.
